# Use of blood-based neurofilament light chain as an endpoint in clinical trials of neurodegenerative conditions: a scoping review

**DOI:** 10.1007/s00415-026-14007-5

**Published:** 2026-07-20

**Authors:** Yuming Zheng, Oneil G. Bhalala, Kai Sin Chin, Rosie Watson, Nawaf Yassi

**Affiliations:** 1 Melbourne Medical School, The University of Melbourne, Parkville, VIC Australia; 2 Department of Medicine, Royal Melbourne Hospital, Parkville, VIC Australia; 3 Genetics and Gene Regulation Division, The Walter and Eliza Hall Institute of Medical Research, Parkville, VIC Australia; 4 Melbourne Brain Centre, The Royal Melbourne Hospital, University of Melbourne, Parkville, Australia; 5 Department of Aged Care, The Royal Melbourne Hospital, Parkville, VIC Australia; 6 Florey Institute of Neuroscience and Mental Health, University of Melbourne, Parkville, Australia

**Keywords:** Neurofilament light, NfL, Blood biomarkers, Serum biomarkers, Plasma biomarkers, Clinical trials, Neurodegenerative diseases

## Abstract

**Introduction:**

Neurofilament light chain (NfL) is a structural axonal protein measurable in CSF and blood, increasingly investigated as a biomarker of neuroaxonal injury in clinical and research contexts. This review aims to explore the use of blood-based NfL as an endpoint in clinical trials of neurodegenerative conditions.

**Method:**

A database search of MEDLINE and EMBASE was conducted to identify interventional clinical trials and/or related post hoc analyses for neurodegenerative diseases, published between 2013 and 2024 that reported the use of serum or plasma NfL as an endpoint. Additional studies from reference lists of included trials were manually considered for inclusion where relevant. Data were charted descriptively by disease type and summarised.

**Results:**

49 studies were included, 29 in multiple sclerosis (MS), eight in amyotrophic lateral sclerosis (ALS), six in Alzheimer’s disease (AD), and six in other diseases. Across studies, reductions in NfL often paralleled improvements in primary efficacy outcomes, supporting its use as a biomarker of disease activity and treatment response. However, several studies demonstrated a lack of concordance between change in NfL and in clinical outcomes, some of which may be related to the non-disease-modifying mechanisms of the interventions studied. This necessitates careful consideration when applying blood-based NfL as a biomarker endpoint for studies involving such interventions.

**Conclusion:**

Blood NfL is a promising biomarker with potential utility as a surrogate endpoint in neurological clinical trials, particularly for diseases with active axonal injury. Further validation, particularly around disease- and intervention-specific interpretation, is needed before blood NfL can be incorporated more routinely as a clinical endpoint.

**Supplementary Information:**

The online version contains supplementary material available at 10.1007/s00415-026-14007-5.

## Introduction

Neurofilaments are important protein constituents of the cytoskeleton in the neuronal axon [1]. They consist of four subunits including neurofilament light chain (NfL), neurofilament medium chain, neurofilament heavy chain, and α-internexin or peripherin [2]. Axonal injury results in extracellular release of neurofilaments, which can be detected in cerebrospinal fluid (CSF) and blood as a biomarker for a variety of neurological diseases [3].

In recent decades, biomarkers have attracted increasing interest in the design of clinical trials targeting neurological diseases [4]. They may offer opportunities to stratify patients by diagnostic subgroups, demonstrate therapeutic target engagement, and assess efficacy and safety in a quantifiable way that complements traditional clinical endpoints [5]. The development of assays such as enzyme-linked immunosorbent assay (ELISA), electrochemiluminescence and fourth-generation single-molecule array (Simoa) technology led to progressive improvement in the sensitivity of blood NfL quantification [6, 7]. Whilst plasma and serum NfL levels are commonly 40 fold or more lower than in CSF, several studies have demonstrated strong correlations between NfL levels across biofluids in multiple neurological diseases [8–12]. The commercial availability of assay kits for NfL, as well as validation of cross-fluid correlation, have promoted rising interest in the exploration of blood NfL as a biomarker in clinical and research settings [13].

These developments have supported growing interest in the use of blood NfL in clinical trials. Recent reviews have comprehensively summarised the biological characteristics of NfL, assay methodologies, and its diagnostic, prognostic and disease-monitoring applications across neurological diseases [14–16]. However, comparatively little attention has been given to the specific use of blood NfL as an endpoint in interventional clinical trials. In this context, a scoping review was undertaken to broadly map how blood NfL has been incorporated across the recent trial literature, and to describe how NfL findings have related to clinical endpoints. This scoping review aims to synthesise current evidence regarding the use of blood NfL as a clinical trial endpoint in clinical trials of neurodegenerative conditions over the past decade, with a focus on trials investigating interventions for neurodegenerative diseases including multiple sclerosis, motor neuron disease, and Alzheimer’s disease, where NfL has been most extensively validated as a longitudinal marker of ongoing axonal injury. Specifically, we sought to characterise how and in what contexts blood NfL has been incorporated as an endpoint in clinical trials, and to describe its concordance with clinical endpoints.

## Methods

This scoping review is reported in accordance with the Preferred Reporting Items for Systematic Reviews and Meta-analyses extension for Scoping Reviews (PRISMA-ScR) (Supplementary Figure S1) [17].

### Search methods

A database search was conducted in MEDLINE and EMBASE, using the following pre-defined search terms “neurodegenerative disease” OR “Parkinson Disease” OR “Alzheimer Disease” OR “dementia” OR “lewy body disease” OR “frontotemporal dementia” OR “amyotrophic lateral sclerosis” OR “motor neuron disease” OR “Huntington disease” OR “spinal muscular atrophy” OR “spinocerebellar ataxia” OR “multiple sclerosis” OR “neuropathy” AND “plasma” OR “serum” OR “blood” AND “neurofilament” OR “NfL” AND “clinical trial”, entered as both MeSH term and Keyword. The search result was limited to English language, human studies, between year 2013 and date of search (8th of February 2024), and excluding conference abstracts. Search terms are included in Supplementary Figure S2.

Additional studies from reference lists of included trials were manually screened and considered for inclusion where relevant.

Journal articles were examined by three independent reviewers through the Covidence systematic review software [18], with discrepancies resolved through consensus.

### Inclusion and exclusion criteria

Inclusion criteria for studies included (1) clinical trials for a neurodegenerative condition; (2) using plasma NfL (pNfL) or serum NfL (sNfL) as a primary, secondary or exploratory endpoint. Exclusion criteria included (1) observational study design; (2) study being protocol only; (3) systematic reviews, posters, or conference abstracts; (4) study using CSF-based NfL without blood-based NfL.

### Data extraction

Extracted data included disease detail, study design, study phase, participant demographics, sample size, blood NfL source, intervention and comparator, treatment duration, primary efficacy or clinical endpoint, primary endpoint findings, and blood NfL findings. Included studies were grouped by disease types. Conditions with more than three included studies were presented through the narrative synthesis. For brevity, conditions represented by three or fewer studies were not discussed in detail.

## Results

**Fig. 1:**
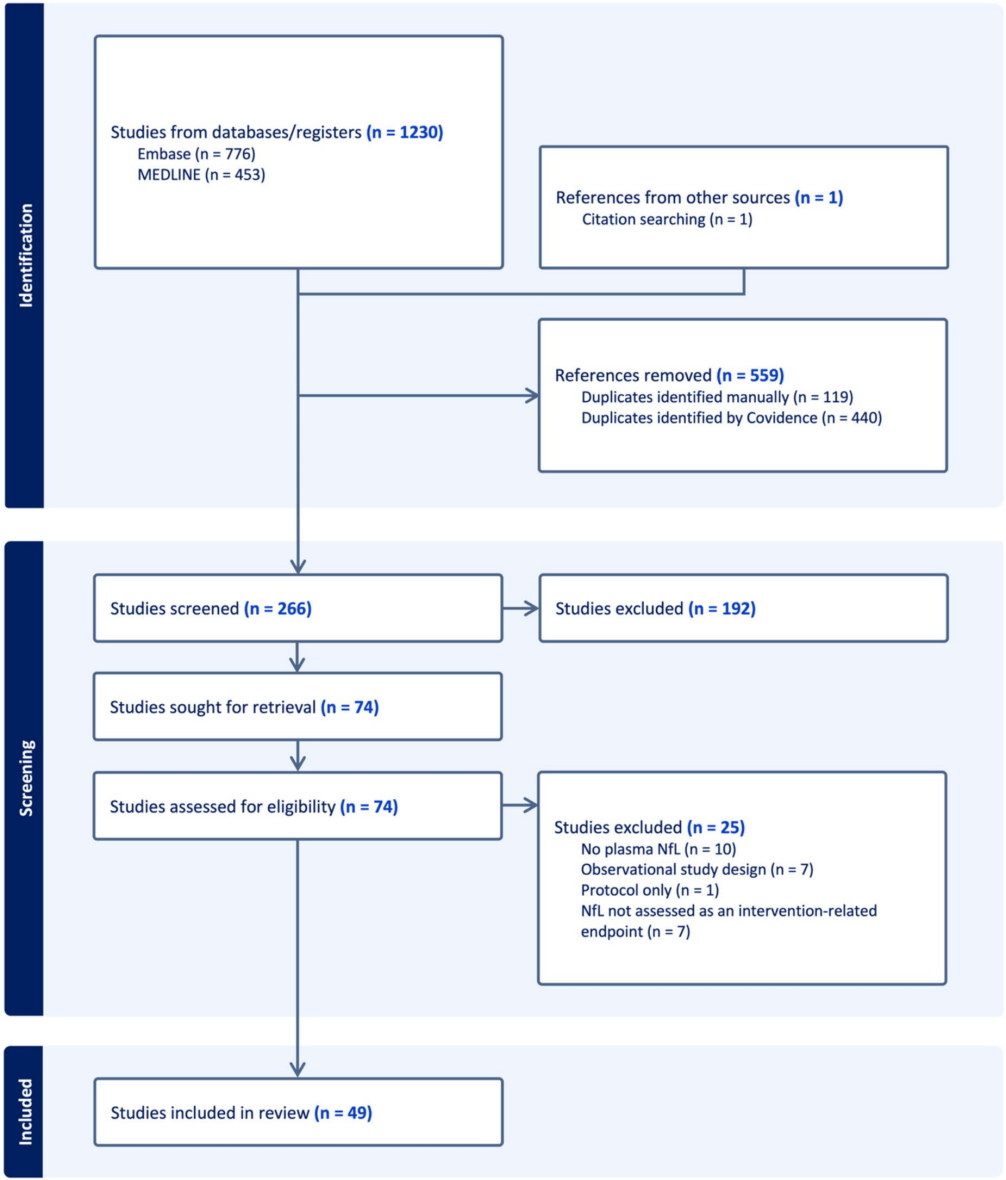
Flowchart summarising the process of study selection. Generated using Covidence [18]

The database search generated 265 unique studies, of which 48 studies were included based on eligibility criteria (Fig. 1). One additional study was found from reference lists of included trials and included for its relevance in AD [19], resulting in 49 included studies overall. Among the 49 included studies, 29 studies investigated multiple sclerosis (MS), eight studies investigated amyotrophic lateral sclerosis (ALS), six studies investigated Alzheimer’s disease (AD), and six studies investigated other neurological diseases, including hereditary transthyretin-mediated amyloidosis with polyneuropathy (hATTR-PN), HIV-associated neurocognitive disorders, acute axonal damage related to bortezomib for multiple myeloma, neuromyelitis optica spectrum disorder (NMOSD), progressive supranuclear palsy (PSP), and multiple system atrophy (MSA). For brevity, the six studies on neurological diseases other than MS, ALS, and AD are not discussed in details in this review. The characteristics of included studies are summarised in Supplementary Table S1. Some publications are represented by multiple entries because they reported results from more than one distinct clinical trial.

Blood NfL was measured using a range of analytical platforms. Simoa-based assays were the most commonly used (38 studies), whilst five studies used ELISA. Six studies did not clearly report the analytical platform used. Serum and plasma were used as the source biofluids for NfL measurement across studies, with no included studies measuring whole blood. Throughout this review, the term "blood NfL" is used when referring collectively to serum and plasma NfL. Where individual studies are discussed, the specific biofluid measured is specified and is also summarised in Supplementary Table S1.

Overall, 20 of the 49 included studies demonstrated concordant positive findings, with statistically significant improvement in both blood NfL and the primary efficacy outcome. Nineteen studies demonstrated concordant neutral findings, six demonstrated discordant findings, and four could not be classified because they primarily evaluated safety or used blood NfL for purposes other than assessing treatment efficacy. A summary of study classifications by disease category is presented in Table 1.

**Table 1:** Classification of included studies according to the relationship between changes in blood neurofilament light chain (NfL) and the primary efficacy outcome

Disease category	Concordant (positive)	Concordant (neutral)	Discordant	Not classifiable
Multiple sclerosis	16 (33%)	9 (18%)	3 (6%)	1 (2%)
Amyotrophic lateral sclerosis	0 (0%)	4 (8%)	2 (4%)	2 (4%)
Alzheimer's disease	2 (4%)	3 (6%)	1 (2%)	0 (0%)
Other neurodegenerative diseases	2 (4%)	3 (6%)	0 (0%)	1 (2%)

Values are presented as number of studies (percentage of all included studies, *n* = 49). Concordant positive indicates statistically significant improvement in both the primary efficacy outcome and blood NfL. Concordant neutral indicates no statistically significant improvement in either the primary efficacy outcome or blood NfL. Discordant indicates statistically significant improvement in either the primary efficacy outcome or blood NfL, but not both. Not classifiable includes studies that primarily evaluated safety or tolerability, or used blood NfL for purposes other than assessing treatment efficacy

### Multiple sclerosis

Overall, twenty-nine of the included studies investigated the effect of interventions on blood NfL level in patients with MS. Concurrent statistically significant change in NfL level and the primary efficacy outcome were seen in 16 studies [20–35]. Three studies detected a statistically significant change in the primary efficacy endpoint without significant change in NfL level [36–38]. Nine studies reported no statistically significant change in either outcome [39–47]. One study [48] did not comment on the effect of an intervention on blood NfL, but rather used blood NfL as a prognostic indicator following intervention.

Five studies explored the effect of lifestyle interventions on blood NfL, two of which investigated the effect of a ketogenic diet [23, 36], the remaining three investigated vitamin D3 supplementation [45–47]. The ketogenic diet studies yielded mixed findings. In one cohort analysed by Bock et al., clinically significant improvement in health-related quality of life was seen in both the caloric restriction group and adapted ketogenic diet group, but only the latter group demonstrated a statistically significant reduction in NfL [23, 49]. In contrast, a single-arm, open-label study analysed by Oh et al. found statistically significant improvement in some clinical outcomes such as expanded disability status scale (EDSS) and 9 hole peg test (9HPT), but no statistically significant change in serum NfL (5.45 pg/mL at baseline, 5.49 pg/mL at 6 months) [36, 50]. The authors attributed this lack of change in NfL to the low baseline levels in study participants (5.45 pg/mL vs 15.17 pg/mL in a historical untreated MS control group), due to their clinically and radiologically stable state of disease, where 97% of participants were on a disease modifying therapy (DMT). The authors highlighted this as one of the potential reasons that the NfL outcome in their study was different to the former cohort studied by Bock et al. [23], who had a higher baseline NfL value, as 29% of participants were untreated and 48% were on low-efficacy DMTs. The divergent results observed suggest that background DMTs may act as a confounder when using NfL to study nutritional impact on MS activity. This warrants further consideration in future trials. All three studies that investigated the effect of vitamin D3 supplementation yielded no statistically significant inter-arm difference in either primary efficacy outcomes or blood NfL [45–47].

Two studies [24, 41] investigated the effect of natalizumab, a monoclonal antibody inhibitor of the α4 subunit of α4β1 and α4β7 integrins, which reduces inflammation primarily through disruption of interaction between lymphocyte integrins and endothelial receptors that enable lymphocytic migration to the CNS [51, 52]. One of these studies, a post hoc analysis of the phase 3 randomised controlled trial (RCT) AFFIRM, demonstrated statistically significant associations between receiving natalizumab and low sNfL level (< 97.5th age-normative percentile) under various temporal definitions in the study (odds ratio (OR) ranging from 4.5 to 6.1). This was consistent with the improvement in clinical outcomes (rate of relapse at 1 year and sustained disability progression at 2 years) seen with natalizumab in the original study [24, 52]. In contrast, an analysis of the phase 3 RCT ASCEND evaluated the longitudinal level of serum glial fibrillary acidic protein (GFAP) which had weak to moderate correlations with sNfL among participants [49, 50, 53], at different time points (estimated Spearman’s correlations: 0.21, 0.30, 0.17 for the natalizumab group; 0.31, 0.33, 0.27 for the placebo group; at weeks 0, 48, 96 respectively). The authors did not specify longitudinal levels of sNfL; however, since sGFAP was stable in both arms throughout the study with no significant inter-arm difference, it was likely that natalizumab did not produce significant change in sNfL. This would be consistent with the lack of treatment effect on the primary efficacy outcome in part 1 of the ASCEND trial, the overall confirmed disability progression (44% in natalizumab group vs 48% in placebo group; OR 0.86; *P* = 0.287) [41, 53].

Four studies investigated the effect of anti-CD20 monoclonal antibodies, including ofatumumab [21, 30], rituximab [39], and ocrelizumab [22]. Two simultaneous phase 3 RCTs, ASCLEPIOS I and II, demonstrated superior effects of subcutaneous ofatumumab on relapsing MS, over oral teriflunomide, producing lower adjusted annualised relapse rate (ARR) (0.11 and 0.10 in ofatumumab vs 0.22 and 0.25 in teriflunomide in ASCLEPIOS I and II, respectively) and better radiological outcomes on MRI. The inter-arm difference was also apparent in the sNfL concentrations, where the level in ofatumumab group was 7% and 11% lower at 3 months, 27% and 26% lower at 12 months, 23% and 24% lower at 24 months, in ASCLEPIOS I and II, respectively [30]. Similar findings were found in the post hoc analysis of the open-label phase 2 trial, APLIOS, which demonstrated consistent decline from baseline to week 12 in sNfL for both groups receiving ofatumumab (via autoinjector or pre-filled syringe) [21, 54]. Treatment with ocrelizumab in the OPERA I and II [55] and ORATORIO [56] trials also produced significant reductions in blood NfL compared to interferon β−1a or placebo, respectively [22]. Among participants of the ORATORIO trial, there was a statistically significant difference in reduction in pNfL from baseline, between the ocrelizumab group (20.2%) and the placebo group (6.7%) at 120 weeks. Similarly in participants of the OPERA I and II trial, there was a statistically significant difference in reduction in sNfL from baseline, between the ocrelizumab group (43.7%) and the interferon group (30.2%) at 96 weeks. This finding in blood NfL was consistent with the positive findings in the primary outcomes of both trials, where ocrelizumab produced a lower ARR [55] and a lower percentage of patients with confirmed disability progression at 12 weeks [56]. In contrast, a study comparing the effect of rituximab and ocrelizumab in patients with primary progressive MS demonstrated no statistically significant inter-arm difference in the primary outcome of time to confirmed disability progression, and no statistically significant difference in sNfL at the end of follow-up [39].

Four studies investigated the effects of sphingosine 1-phosphate modulators, fingolimod [27, 28, 48] and ozanimod [29]. All 3 studies involving fingolimod obtained data from the FREEDOMS [57] and/or TRANSFORMS [58] trials. In both, fingolimod produced lower ARR than the control, along with MRI results supporting the primary outcome. Greater reductions in pNfL concentration were seen in the fingolimod group, when compared to the placebo group at 6 months (35.4% vs 9% reduction from baseline) and 24 months (43% vs 4% reduction from baseline) in the FREEDOMS trial, or the IFN group at 6 months (36% vs 14% reduction from baseline) and 12 months (39% vs 17% reduction from baseline) in the TRANSFORMS trial [27]. Another analysis supported this finding and demonstrated how the mean and median pNfL concentrations at 6 months were lower in the fingolimod group than the placebo group in the FREEDOMS trial, at 31.9 pg/mL vs 23.1 pg/mL and 18 pg/mL vs 26 pg/mL respectively [28]. The authors additionally explored the potential utility of NfL as a primary endpoint for future phase 2 studies, by demonstrating the association between NfL levels at 6 months with other markers of disease activity at 24 months, such as number of relapses (*r* = 0.25, *P* < 0.001), cumulative risk of 6-month confirmed disability worsening (CDW) (HR = 1.83, *P* = 0.012), cumulative number of active lesions (*r* = 0.46, *P* < 0.001), and percentage brain volume change (*r* = − 0.41, *P* < 0.001). However, there was no or weak correlation between change in pNfL at 6 months from baseline and disease parameters at 24 months.

Another analysis focussed on the utility of pNfL as a prognostic predictor for long term outcome, in patients receiving fingolimod 0.5 mg daily, across the FREEDOMS and TRANSFORMS trials [48]. Longitudinal pNfL values, calculated as geometric means over 12 or 24 months, had stronger predictive value for disease progression than baseline pNfL. Elevation in longitudinal pNfL over 24 months had stronger correlation than longitudinal pNfL over 12 months, than baseline pNfL value, with hazard of reaching EDSS ≥ 4.0 (HR = 7.91 vs 2.78 vs 2.19). Baseline NfL was reported as not predictive of risk in reaching 6-month CDW (HR = 1.54, *P* = 0.1059) or 20% worsening in timed 25 foot walk (T25FW) (HR = 1.06, *P* = 0.7988), 9HPT (HR = 1.92, *P* = 0.0695), and paced auditory serial addition test (PASAT) (HR = 1.48, *P* = 0.3559). Whilst elevation in longitudinal pNfL over 12 months was predictive of 20% worsening in PASAT score (HR = 2.59, *P* = 0.0410), elevation in longitudinal pNfL over 24 months was predictive of accelerated 6-month CDW (HR = 3.14, *P* = 0.0061) and 20% worsening in the T25FW (HR = 3.05, *P* = 0.0056). It is interesting to note that there were more patients categorised as having high pNfL at baseline (*n* = 110) than longitudinally over 12 months (*n* = 61) and 24 months (*n* = 22). Presumably, a significant proportion of patients with high baseline pNfL demonstrated a reduction of pNfL in response to therapy. Therefore, it is possible that the correlation between elevated longitudinal pNfL and risk of disease progression reflects prognostic prediction for patients who do not respond to therapy. The authors found that the prognostic model made using longitudinal pNfL over 24 months had a greater area under the ROC (receiver operating characteristic) curve (AUC) than models built using longitudinal pNfL over 12 months or baseline pNfL. Furthermore, the model with the greatest AUC, and hence prognostic value, is one built using a combination of clinical parameters, MRI findings, and longitudinal pNfL over 24 months.

A post hoc analysis on the SUNBEAM [59] and RADIANCE [60] trials evaluated the effect of ozanimod, another sphingosine 1-phosphate modulators, compared to interferon β−1a [29]. In both trials, both dosages of ozanimod (0.46 mg and 0.92 mg) produced statistically significantly better primary outcomes (lower adjusted ARR) compared to interferon. For the SUNBEAM trial, rate ratios of ARR in IFN vs ozanimod 0.46 mg = 0.69, *P* = 0.0013; IFN vs ozanimod 0.92 mg = 0.52, *P* < 0.0001. For the RADIANCE trial, rate ratios of ARR in IFN vs ozanimod 0.46 mg = 0.79, *P* = 0.0167; IFN vs ozanimod 0.92 mg = 0.62, *P* < 0.0001. This is mirrored by the findings in pNfL in the post hoc analysis, where in both trials, both dosages of ozanimod produced statistically significantly greater reduction in pNfL compared to NfL. In the SUNBEAM trial, median percentage change in pNfL = − 13.4% in IFN, − 22.8% in 0.46 mg ozanimod (*P* = 0.0003 compared to IFN), and − 26.9% in 0.92 mg ozanimod (*P* < 0.0001 compared to IFN). In the RADIANCE trial, median percentage change in pNfL = − 15.5% in IFN, − 19.7% in 0.46 mg ozanimod (*P* = 0.0024 compared to IFN), and − 23.5% in 0.92 mg ozanimod (*P* < 0.0001 compared to IFN).

In addition to the ketogenic diet trial discussed above [36], two additional studies demonstrated a lack of concordance between primary endpoint and NfL in response to intervention [37, 38]. In a phase 2 trial of ibudilast, an inhibitor of macrophage migration inhibitory factor and phosphodiesterases 3, 4, 10, and 11 [61, 62], there was no significant difference in sNfL between groups receiving ibudilast and placebo, despite the statistically significant inter-arm difference in rate of change in brain parenchymal fraction over 96 weeks reported in the original trial [33, 34, 37, 59, 60, 61, 63, 34]. The authors proposed an explanation for the discrepancy between the NfL outcome and primary outcome, where ibudilast acted via protection against neuronal deterioration in existing lesions, rather than acting as an anti-inflammatory, based on its lack of ability to reduce development of new inflammatory brain lesions shown in other studies for relapsing or progressive MS [63, 65]. Similarly, an analysis demonstrated no evidence of treatment effect for simvastatin on NfL in patients with SPMS, despite the original trial meeting its primary endpoint, where simvastatin produced significantly lower annualised rate of whole brain atrophy on serial volumetric MRI than placebo (0.288% vs 0.584%) [38, 66]. The authors highlighted the similarity in the finding of this study and that reported by Fox et al. [37], and provided 2 rationales to explain the discrepancy between endpoints: 1. simvastatin acting on possible pathophysiological processes in SPMS independent of neuroaxonal injury; 2. neuroprotection produced by simvastatin was insufficient to cause change in sNfL, the latter of which the authors speculated to be due to the relationship between NfL and neuroinflammation, which simvastatin may not target, as it does not produce systemic immunomodulating effects in this patient group [66].

Across the studies, there are important patterns of correlation between baseline blood NfL and disease activity. Studies showed correlation between high baseline blood NfL and high baseline activity or burden of disease, either clinically or on imaging [17, 19, 24, 26, 28, 31, 35, 37, 42]. Bar-Or et al. [19] and Kuhle et al. [28] additionally reported a correlation between higher baseline NfL and shorter time since last relapse. Baseline NfL has also been shown to correlate with risk of disease progression [19, 24, 31, 44, 45], and risk of clinical or radiological relapse [18, 23, 24, 26].

Another important finding to note is the positive correlation between baseline NfL and age in MS patients (Spearman *R* = 0.356; *P* = 0.03) [47]. This is consistent with the known association between age and NfL reported in a number of studies across a variety of neurological diseases including normal ageing in healthy controls [11, 67–70]. It is also interesting to note that some of the other studies included in this review reported no significant correlation between baseline NfL and demographic factors such as sex, age, age of MS onset, and MS subtypes [34, 35, 44]. Therefore, it is important to consider whether demographic factors, especially age, may be acting as covariates when using NfL as a biomarker in clinical studies for MS.

### Amyotrophic lateral sclerosis

Eight of the included studies investigated the effect of interventions on the blood NfL level for patients with ALS. All eight studies investigated different pharmacological interventions. In three studies, interventions did not produce statistically significant inter-arm difference for change in the primary outcome and change in blood NfL levels [71–73]. In a single-arm trial, ibudilast failed to produce statistically significant change in sNfL and the primary endpoint, the standard uptake value ratio for PBR28-PET in primary motor cortices [74]. Two studies investigated the safety and tolerability of pharmacological agents, and included blood NfL as an exploratory biomarker [75, 76]. In the two remaining studies, there was a lack of concordance between statistical significant change in the primary outcome and in NfL [77, 78].

A key study to note is the phase 3 VALOR RCT, which assessed the effect of tofersen, an antisense oligonucleotide designed to decrease superoxide dismutase 1 (SOD1) protein synthesis, in patients with SOD1 mutated ALS [78]. Over 28 weeks, pNfL level reduced significantly in the tofersen group, with a 60% reduction in the faster progression subgroup, and remained low during the subsequent 24-week open-label extension. By contrast, pNfL levels increased in the placebo group during the initial 28 week of the VALOR trial, with a 20% increase from baseline in the faster progression subgroup. This was followed by a decline in the subsequent 24 weeks of the open-label extension with tofersen treatment, to levels comparable to the tofersen group. It is important to note that tofersen was not able to produce a statistically significant improvement in the primary outcome, the change in revised amyotrophic lateral sclerosis functional rating scale (ALSFRS-R) total score from baseline to week 28, in comparison to the placebo group (− 6.98 in tofersen group versus − 8.14 in placebo group; *P* = 0.97). Following OLE, statistically significant differences in clinical endpoints were seen between early-start and delayed-start cohorts at 52 weeks, where the early-start cohort achieved a slower decline in ALSFRS-R total score by 3.5 points (CI 0.4–6.7), slow vital capacity by 9.2 percentage points (CI: 1.7–16.6) and hand-held dynamometry by 0.28 points (CI: 0.05–0.52). Based on the findings of NfL reduction with treatment, tofersen was approved by the FDA under the accelerated approval pathway for the treatment of SOD1 mutated ALS [79]. Findings from the VALOR trial and OLE highlight the potential of NfL as a surrogate marker of disease activity, particularly in enabling earlier assessment of treatment efficacy in trials, when clinical benefits may take longer to emerge.

Two studies investigated the safety and tolerability of their respective intervention, and used blood NfL as an exploratory biomarker [75, 76]. In a phase 1b open-label dose-escalation trial of atibuclimab, a monoclonal antibody against CD14, sNfL decreased in five of ten patients at day 5 and remained reduced in four at day 33. However, three patients had increased sNfL at day 5, though the patient with the highest increase had their NfL level returned to baseline by day 33. The authors stated that the limitation in sample size and treatment duration may hinder conclusions on relationship between intervention dose and biomarkers. The authors also noted that the trial was not designed to demonstrate efficacy and reported no significant change in clinical parameters such as ALSFRS-R [75]. In a phase 1 first-in-human study, the dual leucine zipper kinase inhibitor GDC-0134 was associated with a reversible, dose-dependent increase in pNfL over the first 12 weeks of the OLE phase of the study [76]. The authors suggested that the rise could be a potential on-target drug effect of dual leucine zipper kinase inhibition. However, they additionally commented that the rise was of concern. This would be consistent with the clinical safety profile of GDC-0134 which was deemed unacceptable, leading to discontinuation of drug development.

Another study to note is a single centre, three-arm (placebo, 1 MIU, 2MIU), double-blinded RCT of aldesleukin, a recombinant analogue of interleukin-2 [77]. The primary endpoint, an increase in proportion of regulatory T cells, was met by both 2MIU and 1MIU arms. The authors reported no drastic increase in the mean pNfL level in the two treatment groups compared to baseline at day 85, and an over 20% increase in mean pNfL level in the placebo group, which authors deemed consistent with disease progression. This increase in the placebo group was, however, not statistically significant. Additionally, there was no statistically significant change in pNfL in the overall population between day 85 and baseline. The authors proposed that the divergence between primary outcome findings and NfL findings may be an issue of power, and additionally proposed that CSF NfL levels may be more informative [12, 80].

### Alzheimer’s disease

Six of the included studies investigated the application of blood-based NfL as a trial endpoint in AD. Two studies investigated exercise-based interventions [81, 82]. In both studies, the intervention failed to produce a statistically significant improvement in the primary efficacy clinical outcome and in blood NfL, when compared to the control arm [81–84]. Four studies [19, 85–87] investigated pharmacological interventions, producing varying responses in primary outcomes and blood NfL, one of which showed no significant change in either [85].

In an open-label phase 2a trial investigating the effect of PTI-125, a small molecule agent that binds and reverses an altered conformation of the scaffolding protein filamin A, in 13 mild to moderate AD patients, statistically significant reduction in a range of CSF and plasma biomarkers was seen, including CSF and plasma NfL at the end of study (day 28) by 22% and over 10%, respectively, compared to baseline [87].

In the double-blinded phase II RCT TRAILBLAZER-ALZ, donanemab, an antibody specific for the N-terminal pyroglutamate Aβ epitope, produced a statistically significant reduction in change of integrated Alzheimer's Disease Rating Scale from baseline to 76 weeks, compared to placebo (− 6.86 vs − 10.06, *P* = 0.04)[88]. In a secondary analysis of the trial, continued increase in pNfL for both arms from baseline to week 76 was seen (15% in the donanemab group, 19% in the placebo group), with no statistically significant difference between the final pNfL levels the two groups reached (23.86 pg/mL in donanemab group versus 26.05 pg/mL in placebo group, *P* = 0.49) [86]. However, there was significant inter-arm difference in changes in plasma pTau217 and GFAP from baseline to week 76, with a 23% and 12% reduction in the donanemab group, and a 6% and 15% increase in the placebo group, respectively [86]. Additionally, PET imaging demonstrated 85.06 centiloids greater reduction in the donanemab group compared to the placebo group (− 84.13 vs 0.93 centiloids) at 76 weeks [88]. The authors proposed two explanations for the lack of concordance between change in pNfL and change in other outcomes: 1. NfL reflected processes of neuronal injury independent or downstream of amyloid pathology [89]; 2. there was a time lag between amyloid pathology and neurodegeneration, causing the NfL level at 76 weeks to reflect amyloid pathology at baseline, rather than change in amyloid pathology in response to intervention.

Lecanemab, another anti-amyloid therapy, is a humanised IgG1 monoclonal antibody against the Aβ soluble protofibrils. It was tested in patients with early AD in the CLARITY trial [19]. All CSF and plasma biomarkers, including pNfL, demonstrated benefit of lecanemab over placebo at 18 months, with the exception of CSF NfL. Additionally, lecanemab produced better outcomes in key efficacy endpoints including Clinical Dementia Rating – Sum of Boxes (1.21 in lecanemab group vs 1.66 in placebo group at 18 months; *P* < 0.001), PET amyloid burden (− 55.48 centiloids in lecanemab group vs 3.64 centiloids in placebo group, in a substudy analysis at 18 months; *P* < 0.001), and ADAS-cog14 (4.14 in lecanemab group vs 5.58 in placebo group at 18 months; *P* < 0.001), compared to placebo. The authors attributed this lack of concordance between CSF NfL and other endpoints to NfL being less sensitive to neurodegeneration and having a slower time course for change. However, they did not offer an interpretation for the lack of concordance between the change in CSF NfL and in plasma NfL, the latter of which was statistically significantly lower in the lecanemab group at 18 months and thus aligned with the beneficial effects observed in other endpoints.

### Other neurodegenerative diseases

NfL use was explored in several clinical trials found in the literature search, outside of the field of MS, ALS, and AD. Statistically significant change in both the primary endpoints and NfL levels was seen in NMOSD treated with inebilizumab, an anti-CD-19, B cell depleting antibody, and in hATTR-PN treated with patisiran, a double-stranded siRNA oligonucleotide [90, 91]. In contrast, no statistically significant difference in change in the primary endpoints or in NfL levels between treatment and placebo arms was seen in PSP, MSA, and HIV-associated neurocognitive disorders [92–94].

An additional use of NfL was also seen in its application in multiple myeloma patients receiving bortezomib, a selective reversible proteasome inhibitor, where NfL acted as a surrogate marker for acute axonal damage from intervention [95].

## Discussion

This scoping review specifically examined the use of blood-based NfL as an endpoint in interventional clinical trials investigating neurological diseases. Unlike previous reviews that have primarily focussed on the biological and clinical applications of NfL, our objective was to evaluate how blood NfL has been incorporated as a clinical trial endpoint and how changes in blood NfL relate to conventional efficacy outcomes.

Across the 49 included studies, some patterns are identified, regarding the utility of blood NfL in clinical and research contexts.

### Baseline NfL as a marker of disease activity and predictor of disease relapse and progression

As discussed in a previous segment, several clinical trials in MS demonstrated correlation between high baseline blood NfL and high baseline activity or burden of disease. Other included studies also reported correlation between baseline blood NfL and faster rate of disease progression as measured by some of the pre-specified metrics, in AD [82], ALS [71, 73], and MS [38, 40], as well as faster onset of relapse in MS [26].

Studies demonstrated that elevation in baseline NfL correlated with worse final clinical or radiological outcome in MS [34, 48], worse cumulative radiological outcome in MS [40], shorter survival time in ALS [73], and higher NfL at the end of the study in MS [27, 34].

These findings seem to indicate that higher NfL at baseline reflects a higher level of disease activity or burden, and may be associated with less favourable clinical and radiological outcomes. Baseline blood NfL may therefore warrant further exploration as a variable in future clinical trials, including for cohort characterisation or subgroup analyses.

### Prognostic information from change in NfL

Some of the included studies reported correlation between greater increase in NfL and faster functional decline in AD [82], more clinical or radiological activity of disease in MS [33, 35, 40], and worse cumulative imaging outcome in MS [40]. In patients with multiple system atrophy (MSA), increases in pNfL were associated with worsening UMSARS scores [92].

Some studies reported correlation between decrease in NfL and improvement in clinical outcomes in MS [20] or less disease activity on imaging in MS [33]. A greater reduction in NfL from baseline correlated with better outcomes on imaging and lower adjusted ARR [29]. Change in sNfL was positively correlated with change in contrast enhancing lesion volume and proportion of IL-10+ CD8+ T cells [33].

Two studies reported on correlations involving transient changes in NfL. Both studies reported correlation between transient increase in NfL and clinical or radiological relapse near the NfL increase, in MS [21] and NMOSD [90]. Aktas et al. [90] additionally reported a correlation between increase in sNfL at NMOSD attack and EDSS worsening, demonstrating the potential value of NfL in identifying patients with higher risk of limited post-relapse recovery.

A post hoc analysis, based on data from the FREEDOMS trial [57], assessed the association between pNfL at 6 months with other MS disease parameters at 24 months, and thereby explores the feasibility of using pNfL as a primary endpoint for future phase 2 trials [28]. Statistically significant correlations were found between pNfL level at 6 months and clinical parameters at 24 months, as discussed earlier in this review. However, it is interesting to note that the change in pNfL at 6 months from baseline was reported to have no or only weak correlation with outcomes at 24 months. Similar finding was reported by another group, who found no correlation between change in sNfL and change in clinical outcome metrics or patient reported outcomes, for MS patients enrolled in a diet-based intervention trial[36]. Sejbaek et al. [32] also reported no correlation between elevation in blood NfL at 1 year of treatment with dimethyl fumarate and clinical disease activity in year 1 or 2. The authors also reported on correlations involving NfL in CSF, the elevation of which after 1 year of treatment was associated with elevated risk of relapse and/or new T2-weighted MRI lesions during year 1, but not with disease activity in year 2. Therefore, given the variability in findings across studies, the prognostic value of change in NfL following treatment remains unclear.

Change in blood NfL was also shown to have potential utility as a marker of neurotoxicity of treatment, as a study demonstrated correlation between change in sNfL and dose of busulfan, which was used as a conditioning agent for immunoablation and autologous haematopoietic stem cell transplantation [35].

Taken together, longitudinal measurement of blood NfL may have the potential to serve as an early indicator of biological response to interventions in clinical trials, with possible implications for timely adaptive trial decisions such as early detection of treatment effects and participant safety monitoring in the selected contexts.

### Studies with a lack of concordance between primary outcome and NfL

This review discussed several studies where there is incongruence in statistical significance between change in primary outcome and change in blood NfL, following intervention. Camu et al. [77], Miller et al. [78], and Oh et al. [36] proposed explanations for this dissociation in relation to issues in trial design or characteristics of study cohort. Pontecorvo et al. [86], Fox et al. [37], and Williams et al. [38] proposed explanations related to mechanism of their respective intervention. Fox et al. [37] and Williams et al. [38] proposed that their respective intervention provided clinically beneficial effects through neuroprotection without targeting neural inflammatory, which is known to be associated with NfL [96]. Pontecorvo et al. [86] proposed that the neuronal injury may be independent, downstream, or temporally delayed from pathological processes involving amyloid, which donanemab targets. Among the 20 studies with significant change in blood NfL following intervention, 17 studies involved intervention methods that theoretically exerted anti-inflammatory effects as a part of their mechanisms [21–35, 87, 90]. The remaining three studies investigated agents that protected against neuroaxonal injury through other mechanisms, tofersen reducing synthesis of SOD1 protein [78], patisiran reducing production of transthyretin [91], and clemastine fumarate inducing oligodendrocyte differentiation and re-myelination [20]. It is possible that one of the explanations proposed by Williams et al. [38] is correct, whereby blood NfL may primarily reflect ongoing neuroaxonal injury associated with active neuroinflammatory processes. Consequently, interventions that produce clinically beneficial effects through biological pathways that are not directly coupled to ongoing neuroaxonal injury may improve clinical outcomes despite producing little or no measurable change in blood NfL.

It is interesting to note that in the CLARITY trial, CSF NfL did not detect a beneficial effect of lecanemab over placebo, whilst other clinical, radiological, and fluid-based biomarker endpoints including pNfL did. The authors attributed this discrepancy to NfL being less sensitive to neurodegeneration and having a slower time course of change than other biomarkers. This highlighted an additional consideration when interpreting NfL as a biomarker for trials in neurodegenerative diseases.

However, it is important to consider that some of the studies included in this review where the interventions produced no statistically significant change in blood NfL levels may have been underpowered. Future clinical trials incorporating blood NfL as an efficacy endpoint should therefore consider optimisation of statistical power through study design.

Based on studies included in this review, it appears that blood NfL can reflect patient response to intervention methods that exert anti-inflammatory effects. However, when assessing patient response to non-immunomodulating interventions, there may be a need for careful consideration when applying blood-based NfL as a biomarker endpoint for clinical trials.

In addition, the utility of blood NfL as a biomarker may vary according to the temporal dynamics of axonal injury within different diseases. As discussed in a previous segment, NfL appears to be a more informative biomarker for diseases where neuroaxonal injury accrues or flares acutely within typical trial timeframes, such as ALS, MS, NMOSD, and hATTR-PN, and is less informative in slowly progressive diseases where the clinical trial period makes up only a small proportion of the disease natural history, such as PSP, MSA, HIV-associated neurocognitive disorders, and perhaps to some extent AD.

### Limitations

This scoping review has several limitations. As a scoping review, it was designed to map the current use of blood NfL in interventional clinical trials rather than to formally evaluate its validity as a surrogate endpoint. Consequently, although patterns of concordance and discordance between blood NfL and clinical outcomes were described, this review did not assess blood NfL against established surrogate endpoint validation or regulatory qualification frameworks such as the Prentice criteria [97]. We note this is an area of emerging importance due to the increased use of NfL in clinical trials. Critical appraisal was not undertaken for included studies, consistent with the mapping and exploratory purpose of this review. As a result, differences in study quality may have influenced findings, and formal appraisal would be useful in future reviews seeking to evaluate the strength of evidence for blood NfL as a trial endpoint. Additionally, heterogeneity in NfL assay platforms across studies represents a potential source of methodological variability, although its influence on reported biomarker findings could not be assessed within the scope of this review. The review was also limited by reliance on published reporting of blood NfL outcomes. Some clinical trials may have measured or explored NfL without publishing these findings, meaning the current review may not capture all trial uses of blood NfL. Finally, much of the included literature was concentrated in multiple sclerosis, with fewer studies in ALS, AD, and other neurological diseases. This may limit how readily the patterns observed in this review can be generalised across disease areas.

## Conclusion

This scoping review highlights the growing role of blood NfL as a clinical trial endpoint across neurodegenerative conditions. Blood NfL shows considerable promise as a surrogate marker of disease activity and treatment response, though further validation and careful consideration is needed to define its role across a range of neurological diseases and when applied to clinical studies for interventions impacting diverse mechanisms.

## Supplementary Information

Below is the link to the electronic supplementary material.

Supplementary file1 (PDF 95 KB)

Supplementary file2 (PDF 59 KB)

Supplementary file3 (XLSX 47 KB)

## Data Availability

The datasets used and/or analysed during the current study are available from the corresponding author on reasonable request.

## Abbreviations

9HPT: 9-Hole Peg Test
AD: Alzheimer’s disease
ADAS-cog: Alzheimer's disease Assessment Scale Cognitive section
ALS: Amyotrophic lateral sclerosis
ALSFRS-R: Amyotrophic lateral sclerosis functional rating scale
ARR: Annualised relapse rate
AUC: Area under the curve
CDW: Confirmed disability worsening
CNS: Central nervous system
CSF: Cerebrospinal fluid
DMT: Disease modifying therapy
EDSS: Expanded Disability Status Scale
FDA: Food and Drug Administration
GFAP: Glial fibrillary acidic protein
HHD: Hand-Held dynamometry
IL: Interleukin
MRI: Magnetic resonance imaging
MS: Multiple sclerosis
NMOSD: Neuromyelitis optica spectrum disorder
NfL: Neurofilament light
OLE: Open-label extension
PASAT: Paced Auditory Serial Addition Test
PET: Positron emission tomography
pNfL: Plasma neurofilament light
RCT: Randomised controlled trial
sNfL: Serum neurofilament light
SVC: Slow vital capacity
T25FW: Timed 25-foot walk
UMSARS: Unified Multiple System Atrophy Rating Scale
hATTR-PN: Hereditary transthyretin-mediated amyloidosis with polyneuropathy
