# Supplementary material for: Use of blood-based neurofilament light chain as an endpoint in clinical trials of neurodegenerative conditions: a scoping review
Source: J Neurol. 2026 Jul 20;273(8):476. doi: 10.1007/s00415-026-14007-5 (PMC13385163; doi:10.1007/s00415-026-14007-5)
Supplement: Supplementary file 2 — Supplementary file2 (PDF 59 KB) [file 415_2026_14007_MOESM2_ESM.pdf]

Medline search terms

1.	((neurodegenerat* or neuro-degenerat* or neuro degenerat*) adj2 disease*).mp. [mp=title, abstract, heading word, drug trade name, original title, device manufacturer, drug manufacturer, device trade name, keyword heading word, floating subheading word, candidate term word]
2.	*Neurodegenerative Diseases/
3.	*Nervous System Diseases/ or *Neurology/ or neurolog*.mp.
4.	Parkinson Disease/ or parkinson* disease*.mp.
5.	Alzheimer Disease/ or alzheimer* disease*.mp.
6.	dementia*.mp. or *Dementia/
7.	(lewy body dementia* or lewy body disease*).mp. [mp=title, abstract, heading word, drug trade name, original title, device manufacturer, drug manufacturer, device trade name, keyword heading word, floating subheading word, candidate term word]
8.	Lewy Body Disease/
9.	exp Frontotemporal Dementia/
10.	(frontotemporal dementia* or fronto-temporal dementia* or fronto temporal dementia*).mp. [mp=title, abstract, heading word, drug trade name, original title, device manufacturer, drug manufacturer, device trade name, keyword heading word, floating subheading word, candidate term word]
11.	(amyotrophic lateral sclerosis or ALS).mp. [mp=title, abstract, heading word, drug trade name, original title, device manufacturer, drug manufacturer, device trade name, keyword heading word, floating subheading word, candidate term word]
12.	Amyotrophic Lateral Sclerosis/
13.	(motor neuron* disease* or motor-neuron* disease*).mp. [mp=title, abstract, heading word, drug trade name, original title, device manufacturer, drug manufacturer, device trade name, keyword heading word, floating subheading word, candidate term word]
14.	exp Motor Neuron Disease/
15.	Huntington Disease/ or huntington* disease*.mp.
16.	spinal muscular atrophy.mp. or exp Muscular Atrophy, Spinal/
17.	spinocerebellar ataxia.mp. or exp Spinocerebellar Ataxias/
18.	multiple sclerosis.mp. or exp Multiple Sclerosis/
19.	neuropath*.mp.
20.	(AD or PD or MND or ALS or SMA or SCA or MS).tw.
21.	1 or 2 or 3 or 4 or 5 or 6 or 7 or 8 or 9 or 10 or 11 or 12 or 13 or 14 or 15 or 16 or 17 or 18 or 19 or 20
22.	(neurofilament light chain* or NfL*).mp.
23.	(blood or plasma or serum).mp.
24.	22 and 23
25.	21 and 24
26.	clinical trial*.mp.
27.	25 and 26
28.	limit 27 to english language
29.	limit 28 to human
30.	limit 29 to yr="2013 -Current"
31.	limit 30 to conference abstract status
32.	30 not 31

Embase Search terms

1.	((neurodegenerat* or neuro-degenerat* or neuro degenerat*) adj2 disease*).mp. [mp=title, book title, abstract, original title, name of substance word, subject heading word, floating sub-heading word, keyword heading word, organism supplementary concept word, protocol supplementary concept word, rare disease supplementary concept word, unique identifier, synonyms, population supplementary concept word, anatomy supplementary concept word]
2.	*degenerative disease/
3.	*neurologic disease/ or *neurology/ or neurolog*.mp.
4.	exp Parkinson Disease/ or parkinson* disease*.mp.
5.	Alzheimer Disease/ or alzheimer* disease*.mp.
6.	dementia*.mp. or *Dementia/
7.	(lewy body dementia* or lewy body disease*).mp. [mp=title, book title, abstract, original title, name of substance word, subject heading word, floating sub-heading word, keyword heading word, organism supplementary concept word, protocol supplementary concept word, rare disease supplementary concept word, unique identifier, synonyms, population supplementary concept word, anatomy supplementary concept word]
8.	diffuse Lewy body disease/
9.	exp frontotemporal dementia/
10.	(frontotemporal dementia* or fronto-temporal dementia* or fronto temporal dementia*).mp. [mp=title, book title, abstract, original title, name of substance word, subject heading word, floating sub-heading word, keyword heading word, organism supplementary concept word, protocol supplementary concept word, rare disease supplementary concept word, unique identifier, synonyms, population supplementary concept word, anatomy supplementary concept word]
11.	(amyotrophic lateral sclerosis or ALS).mp. [mp=title, book title, abstract, original title, name of substance word, subject heading word, floating sub-heading word, keyword heading word, organism supplementary concept word, protocol supplementary concept word, rare disease supplementary concept word, unique identifier, synonyms, population supplementary concept word, anatomy supplementary concept word]
12.	amyotrophic lateral sclerosis/
13.	(motor neuron* disease* or motor-neuron* disease*).mp. [mp=title, book title, abstract, original title, name of substance word, subject heading word, floating sub-heading word, keyword heading word, organism supplementary concept word, protocol supplementary concept word, rare disease supplementary concept word, unique identifier, synonyms, population supplementary concept word, anatomy supplementary concept word]
14.	exp motor neuron disease/
15.	Huntington chorea/ or huntington* disease*.mp.
16.	spinal muscular atrophy.mp. or exp spinal muscular atrophy/
17.	spinocerebellar ataxia.mp. or spinocerebellar degeneration/
18.	multiple sclerosis.mp. or exp multiple sclerosis/
19.	neuropath*.mp.
20.	(AD or PD or MND or ALS or SMA or SCA or MS).tw.
21.	1 or 2 or 3 or 4 or 5 or 6 or 7 or 8 or 9 or 10 or 11 or 12 or 13 or 14 or 15 or 16 or 17 or 18 or 19 or 20

22.	((((((plasma or plasma based or plasma-based) adj2 (biomarke* or protei*)) or (blood or blood based or blood-based)) adj2 (biomarke* or protei*)) or (serum or serum based or serum-based)) adj2 (biomarke* or protei*)).mp. [mp=title, book title, abstract, original title, name of substance word, subject heading word, floating sub-heading word, keyword heading word, organism supplementary concept word, protocol supplementary concept word, rare disease supplementary concept word, unique identifier, synonyms, population supplementary concept word, anatomy supplementary concept word]
23.	(neurofilament light chain* or NfL*).mp.
24.	(blood or plasma or serum).mp.
25.	23 and 24
26.	21 and 25
27.	clinical trial*.mp.
28.	26 and 27
29.	limit 28 to english language
30.	limit 29 to human
31.	limit 30 to yr="2013 -Current"
32.	limit 31 to conference abstract status
33.	31 not 32
